# Sleep Differences in Firefighters: Barracks vs. Home

**DOI:** 10.3390/ijerph21091155

**Published:** 2024-08-30

**Authors:** Lainey E. Hunnicutt, Makenzie Corgan, Sarah R. Brown, Alyssa Nygaard, George Lesley Meares, Scott R. Collier

**Affiliations:** Department of Public Health and Exercise Science, Appalachian State University, Boone, NC 28608, USA; hunnicuttle@appstate.edu (L.E.H.); mearesgl@appstate.edu (G.L.M.IV)

**Keywords:** sleep architecture, firefighters, sleep characteristics

## Abstract

It has been shown that the firefighter occupation leads to poor sleep quality and sleep architecture. Disturbed sleep in these occupations can lead to deleterious outcomes including a series of chronic diseases and illnesses such as CVD. Purpose: The aims were (1) to quantify firefighters’ sleep via polysomnography, (2) to identify differences between sleeping in the barracks versus sleeping at home, and (3) to compare firefighter data to age-matched normative data. We expected significant differences between both the home and the barrack conditions as well as significant differences when both conditions were compared to normative data. Methods: 10 male firefighters completed 3 nights of polysomnography recordings (SleepProfiler^TM^ (Advanced Brain Monitoring, Carlsbad, CA, USA)) counterbalanced in both their own beds or barracks. A one-way rmANOVA statistical analysis was used to determine differences in sleep values with a Bonferroni correction if a significant difference was found with significance set at *p* < 0.05. Results: Three important variables, cortical arousals (*p* < 0.05), autonomic activations (*p* < 0.01), and spindle duration (*p* < 0.01), had differences that were statistically significant between sleep at home or in the barracks, with sleep in the barracks being more disturbed. Clinical differences were also observed between the home and barrack conditions and all sleep results were more deleterious when compared to normative data. Conclusions: The data demonstrates that firefighters show poor sleep quality and heavily impacted sleep architecture. This may be due to the effects of rotating shifts and occupational stress on the sleep–wake cycle. These results, when compared to age-matched normative data, show clinical manifestations of disturbed sleep in the firefighter population.

## 1. Introduction

Sleep is a fundamental biological process that profoundly influences various aspects of health. Memory function, metabolic function, and cellular maintenance are among the functions impacted by sleep quality and quantity [1,2]. More specifically, sleep aids in memory consolidation, plasticity, and learning [3]. It is recommended that healthy adults, aged 18 to 64 years, sleep 7 to 9 h a night. Irregular sleep or disrupted sleep has been connected to an increased risk of cardiovascular disease, such as hypertension, impaired adaptive immunity, and decreased neurocognitive performance [2,3]. The quality of sleep is determined by sleep architecture.

Sleep architecture is the progression of sleep stages and cycles throughout the night and gives insight into the quality of sleep [4]. Each cycle is approximately 70 to 120 min long with time spent in each stage lengthening throughout the night [2,5,6]. The cycle consists of two distinct stages: non-rapid eye movement (NREM) and rapid eye movement (REM) [7]. NREM is further divided into three stages (N1, N2, and N3) each with unique characteristics and resulting in deeper sleep from N1 to N3 [8]. Each stage produces a distinctive wavelength, allowing researchers to not only analyze the time spent in each stage but also identify any abnormalities and where in the cycle they may occur. REM and N1 sleep demonstrate high-frequency and low-amplitude waves, similar to those taken while the subject is awake [8]. N3, also referred to as slow wave sleep (SWS), captures low-frequency and high-amplitude waves representing the slowing down of physiological processes [8]. N2 is characterized by the presence of sleep spindles, which are small, isolated bursts of neural activity signaling interactions between the thalamic reticular nucleus and other gray matter in the brain [9]. The differences between stages produce unique attributes that distinguish an individual’s sleep architecture [8]. Electroencephalogram (EEG) recordings can be used to distinguish the different stages and transient arousals during the night. The differences between stages produce unique attributes that distinguish an individual’s sleep architecture.

Certain populations are at an increased risk of sleep disturbance, with shift workers being one of the most prominent. Shift workers contradict their own biological clock leading to disruptions in their sleep–wake cycles [10]. Firefighters are shift workers who have the added obstacle of a change in the sleep environment, moving from sleeping at home to sleeping in the barracks while on shift. A change in sleep environment has been associated with an increase in sleep disturbance [10]. Firefighters also deal with high amounts of occupational stress due to the nature of their work [11]. Individuals with higher levels of stress are more likely to report sleep disturbances or low sleep quality [11]. The combination of shift work, environmental change, and occupational stress creates low sleep quality.

Previous research has shown firefighters sleep poorly [12,13,14]. In one study, firefighters slept significantly less than the US general population [12]. It also showed that firefighters slept longer on nights they were off duty than on nights they were sleeping in the barracks [12]. In other studies, 60% and 73% of the firefighting participants had poor sleep quality [13,14]. The data reported were derived from either actigraphy (using heart rate and movement to determine sleep architecture) or questionnaires rather than using EEG readings.

While studies on shift work and sleep deprivation have been investigated, to this author’s knowledge, there have been no studies examining the differences between sleep environments in firefighters using EEG. Using EEG for the data collection method will minimize the disturbance caused by abnormal lab settings or an increase in uncomfortable equipment. Therefore, the purpose of this study was to elucidate the differences in sleep characteristics between full-time firefighters when sleeping in the barracks and at home, compared to mean age-matched normative values. We hypothesized that firefighters would have changes in REM, NREM, and sleep architecture when on shift vs. off shift due to sleeping in the barracks and being woken for calls throughout the night.

## 2. Materials and Methods

Ten male paid firefighters (18–55 years) from a small, rural community took part in this study. Participants were working a 24 h on and 48 h off rotation schedule from 7 a.m. to 7 a.m. Exclusion criteria included any prior sleep lab experience and/or physician-diagnosed sleep disorders. All participants were familiarized with the sleep profilers and provided informed consent. This study was approved by the University’s Institutional Review Board. The conditions examined were three nights slept in the barracks versus three nights slept at home. The third night of each condition was used in analysis. After compiling our data, we compared sleep at home, sleep in the barracks, and age-matched normative data. Age-matched normative data comes from the National Sleep Foundation [15].

Subjects were given written and verbal instructions on using an Advanced Brain Monitoring SleepProfiler^TM^ (Advanced Brain Monitoring, Carlsbad, CA, USA). This tool collects EEG data to determine sleep architecture and has demonstrated a greater validity and reliability of data collection [16]. The SleepProfiler^TM^ used an algorithm to differentiate between delta, theta, alpha, sigma, beta, and electromyography bands to score sleep patterns [17]. Subjects were directed to maintain regular sleep habits and patterns for three consecutive nights for each data collection period. The study selection was counterbalanced between the conditions at home and in the barracks. Between data collection periods, subjects returned to the lab with the SleepProfiler^TM^, where the data were downloaded and the profiler reprogrammed then returned to the subject to repeat under the other condition.

All data were analyzed for outliers and descriptive statistics were determined for each condition. A repeated measures ANOVA (rmANOVA) was employed to assess the impact of environmental conditions on sleep and any differences in the variables in home vs. barracks. If significance was observed, a Bonferroni correction factor was applied to determine where any differences lay. Due to the small sample size, the authors have reported the mean ± standard error (SE). Statistical significance is characterized by a *p*-value of ≤0.05.

## 3. Results

The sleep profiler used electrodes placed on the foreheads of the participants to monitor and record brain waves from the frontal lobe. This produced measurements such as total sleep time, sleep efficiency, N1, N2, REM and slow wave sleep (N3), wake after sleep onset (WASO), cortical arousals, autonomic activations, and spindle duration. The data were then assessed and statistical analysis was determined using Statistical Package for the Social Sciences, 28, Chicago, IL, USA (SPSS Inc., 28, Chicago, IL, USA). The firefighter data both in the barracks and at home are displayed in Table 1. The age-matched normative values for this population are listed in Table 2.

## 4. Data Collection

As seen in Table 1 and Table 2, firefighters under both conditions had a decrease in time spent asleep when compared to age-matched normative values. Sleep efficiency, however, was in the normal range. There was less time spent in REM sleep and N2 with an increased amount of time in N1 across both conditions. Time in SWS was lower for firefighters while sleeping in the barracks and while they were sleeping at home.

**Figure 1:**
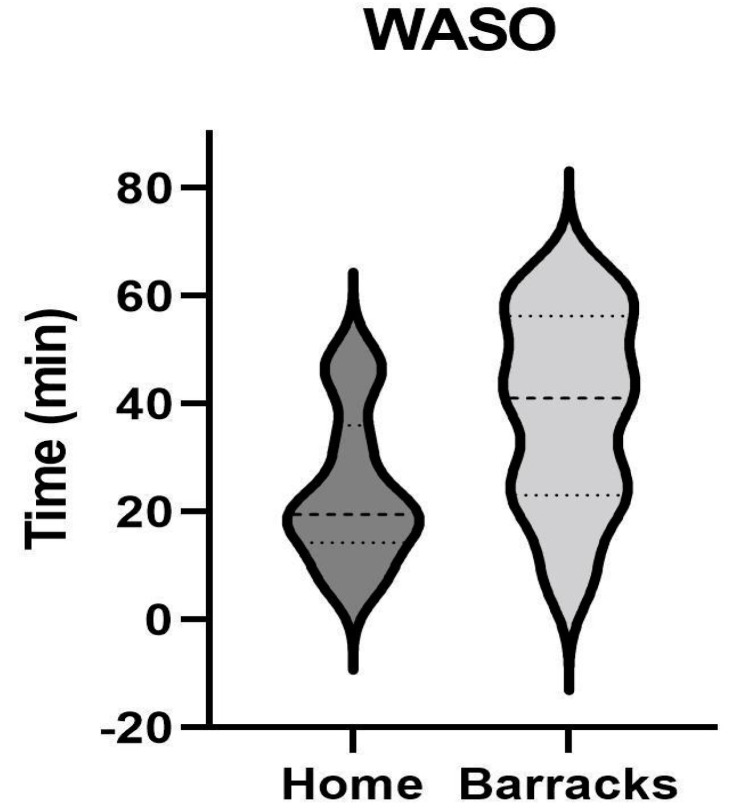
Wake after sleep onset (WASO) in firefighters at home vs. barracks. N = 10. The top and bottom dotted lines represent 25th and 75th percentile respectively. The middle dotted line represents the 50th percentile.

WASO was within the normal range across both conditions. When compared between the home vs. barracks, results were not statistically significant (*p* = 0.056, eta^2^ 0.44), but determined to be clinically relevant, meaning it may still have a detrimental impact on health. Firefighters spent 15 more minutes awake, on average, sleeping in the barracks than when sleeping at home. The differences can be seen in Figure 1.

**Figure 2:**
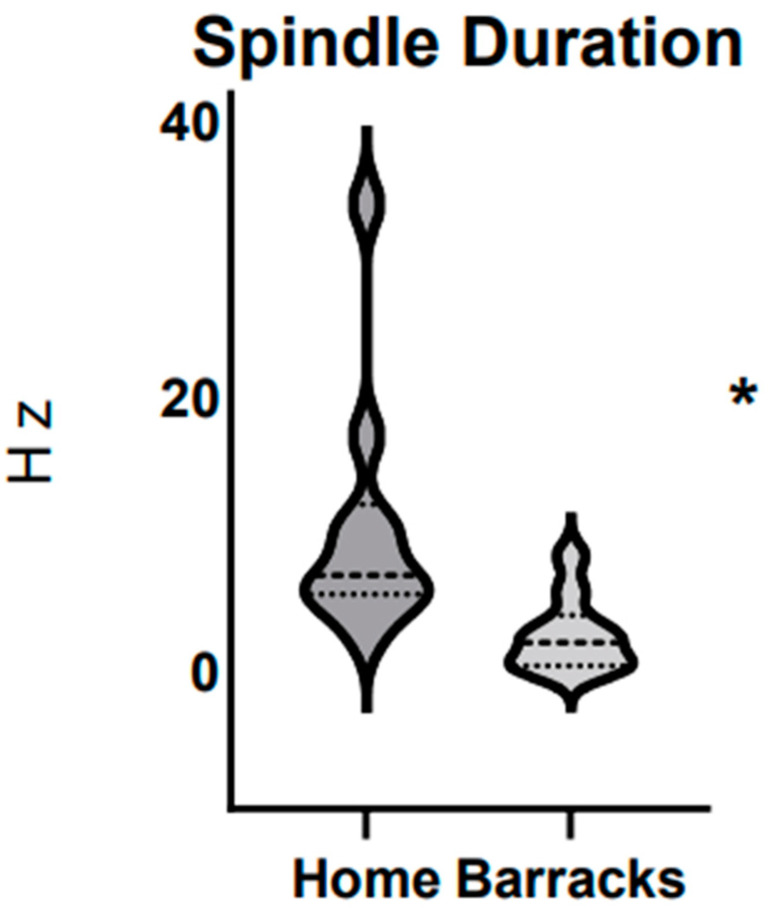
Sleep spindles in firefighters at home vs. barracks. N = 10. Top and bottom dotted lines represent 25th and 75th percentile respectively. The middle dotted line represents the 50th percentile. The * represents a significant difference found between groups.

Sleep spindles were much lower while sleeping in the home condition compared to barracks and the well below normative data. Firefighters averaged 2.6 Hz with a standard of error of 0.85 while sleeping in the barracks. Age-matched data states normal values are between 9 and 15 Hz. Sleep spindles while sleeping at home did fall into this range with an average of 11.0 Hz with a standard error of 2.9. There was a significant difference in sleep spindles between the home and barrack conditions, which can be seen in Figure 2.

**Figure 3:**
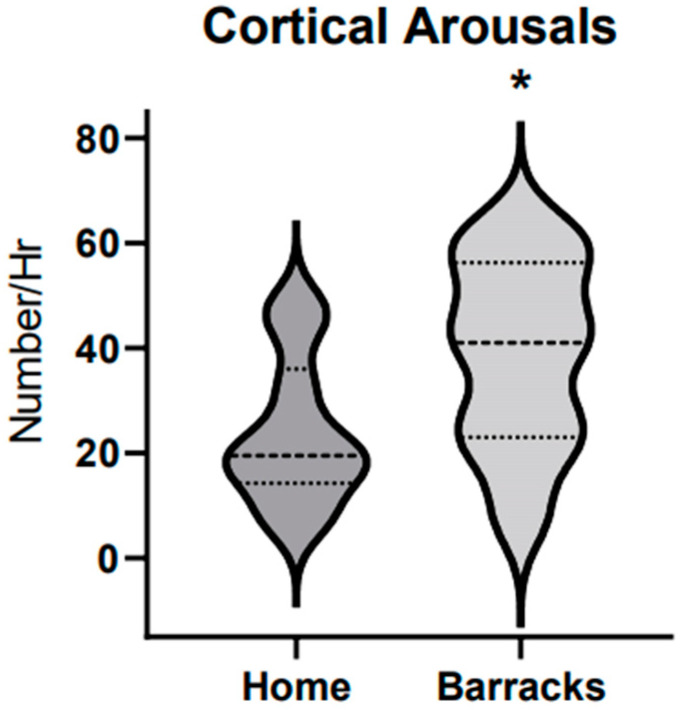
Cortical arousals in firefighters at home vs. barracks. N = 10. Top and bottom dotted lines represent 25th and 75th percentile respectively. The middle dotted line represents the 50th percentile. The * represents a significant difference found between groups.

Cortical arousals in firefighters while sleeping at home and in the barracks were also within the normal range provided by the age-matched normative data. There was significance (*p* < 0.01, eta^2^ 0.56) when compared between the home and barrack conditions. In the barracks, firefighters were being awoken on average 17 times per hour from cortical arousals with a standard error of 2.4 compared to only 12 times an hour with a standard error of 1.7 while sleeping at home. Data are displayed in Figure 3.

**Figure 4:**
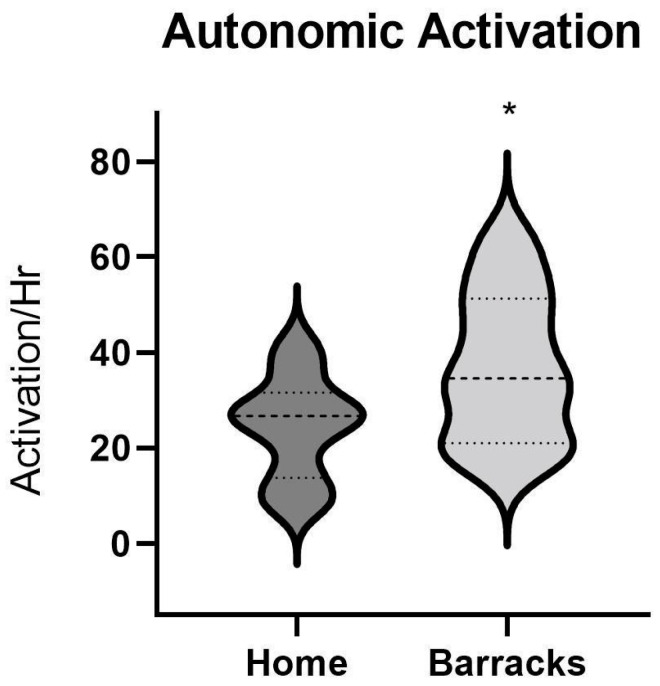
Autonomic activation (AA) in firefighters at home vs. barracks. N = 10. Top and bottom dotted lines represent 25th and 75th percentile respectively. The middle dotted line represents the 50th percentile. The * represents a significant difference found between groups.

Autonomic activation also had a significant (*p* < 0.05, eta^2^ 0.62) when comparing between the home and barrack conditions. While in the barracks, subjects had an average of 37.0 autonomic activations per hour with a standard error of 5.0, while the subjects at home demonstrated an average of 24.0 activations per hour with a standard error of 3.4. Data between conditions are displayed in Figure 4. No age-matched normative data could be found for autonomic arousals.

## 5. Discussion

This study demonstrates that firefighters, regardless of sleeping conditions, are below the average of normative sleep architecture levels. Total sleep time, N2, SWS, and REM were all lower regardless of sleeping conditions when compared to normative values. Despite total sleep time being lower, sleep efficiency remained within normative values. This could be due to the fatigue associated with firefighting. Statistically significant differences were found when comparing the home versus barrack conditions for measurements of cortical arousals, autonomic activations, and spindle duration, and clinical differences were found between conditions for wake after sleep onset and slow wave sleep.

WASO refers to the duration of wakefulness occurring after defined sleep onset and before final awakening [18]. Firefighters spent more time in WASO when at the barracks (39.0 +/− 5.7 min) than they did at home (24.0 +/− 4.4). While the data were within the normative values (0–45 min per night), the National Sleep Foundation states a WASO of < 20 min indicates good sleep quality across all age groups [18]. However, both conditions demonstrated a WASO above 20 min. This value is a way to assess sleep quality and can be used to describe sleep fragmentation. A high WASO value reflects the individual experiencing either longer periods of wakefulness or waking up more frequently during the night. Recently, longer WASO has demonstrated negative cognitive effects, such as worsened working memory [18].

Our result was expected due to the nature of firefighting and the change in sleep environments experienced due to shift work. In a study conducted by Florida Central University (FCU), WASO minutes were significantly different between nights on shift and off shift [12]. On shift, their WASO was 86.8 min, and off shift was 57.7 min (*p* < 0.001) [19]. The difference between studies could be explained by the fact that the FCU study used Sleep Watches rather than the Sleep Profilers^TM^ and had a greater sample size. Sleep watches rely on heart rate and movement to determine data about sleep while the Sleep Profilers^TM^ monitors brain activity. The FCU study was conducted in a larger, more populated city compared to this study conducted in a rural community [12]. A higher WASO can be found in those spending more of their night in lighter stages of sleep such as N1 and N2.

Sleep spindles are characteristic of neural activity in N2 sleep and are related to memory, learning, and synaptic plasticity during sleep [19]. Firefighters experience less time in deeper stages of sleep leading to significantly lower sleep spindles when sleeping in the barracks (2.6 +/− 0.85 Hz) compared to sleeping at home (11.0 +/− 2.9 Hz). Sleep spindles were also lower when sleeping in the barracks when compared to normative data (9–15 Hz). Sleep spindles have been known to play a major role in the conservation of sleep flow while also having a function in overnight retention and memory trace [20]. Though it is not yet fully understood, it can still provide valuable insight into sleep quality.

While both groups of firefighters had cortical arousals within the normal range (NR) (NR 0–20 cortical arousals/hour), these data show a significantly higher instance of cortical arousals when sleeping in the barracks (17 +/− 24 cortical arousals/hour) versus sleeping at home (12.0 +/− 1.7 cortical arousals/hour). This variation could be due to the change in the sleep environment, the sleep environment itself, and the associated stress of being on call. Nocturnal cortical arousals have been linked to fragmented sleep causing drowsiness throughout the day [21]. Those with higher levels of cortical arousals experienced instability in respiratory control, leading to conditions such as sleep apnea and further disruption to sleep architecture [22]. Elevated cortical arousal levels along with the increased likelihood of apneic events in the barracks combine to increase deleterious effects on sleep architecture and affect activities during wakefulness [23,24]. Huang et al. showed a 30% increase in the prevalence of obstructive sleep apnea among firefighters [23].

Sleep architecture is heavily influenced by the interactions between the central and autonomic nervous systems, as well as the cortical and cardiovascular activities related to sleep [24]. In this study, autonomic activations were significantly higher for firefighters sleeping in the barracks (37 +/− 5 autonomic activations/hour) compared to sleeping at home (24 +/− 3.4 autonomic activations/hour). Autonomic activations associated with cortical events caused the neural outflow to the cardiovascular system to fluctuate leading to multiple sleep disturbances [24]. Autonomic activations, alone, can cause cardiovascular consequences, impair daytime functioning, and impact prior cognitive injuries [21].

As hypothesized, autonomic and cortical arousals exhibited a higher occurrence of sleep disturbances in firefighters when sleeping in the barracks vs. home collection. This could be due to the high stress level associated with being on duty. While on shift, firefighters respond to a multitude of calls ranging from car accidents to fires and are often first responders on calls in which police and paramedics are dispatched [11]. Firefighters report high levels of post-traumatic stress and occupational stress [11].

## 6. Conclusions

Deficiencies in sleep quality have been linked to decreases in job performance, poor decision-making, increased stress levels, cardiovascular disease, and psychological disorders [3]. Firefighters experience deficits in sleep architecture regardless of sleep environment, however, these deficits are exacerbated by changes in sleep conditions. Our home vs. barracks data show significant differences in cortical arousals, autonomic activation, and spindle duration that link to poor sleep quality and sleep disturbances.

This is the first study to analyze sleep data in a subject’s own bed using the SleepProfiler^TM^ to capture EEG. Familiarization with the sleep profiler equipment has shown that three nights is ideal for data that are reflective of normal sleep [25].

The methodology of this study was the first of its kind to observe sleep discrepancies in shift work, but the small sample size proved to be a large limitation. Other limitations include not evaluating the association of sleep with memory processing and not measuring breathing events during the night. Future studies are underway aiming to improve and further the results of this study. These studies hope to introduce a third condition made up of volunteer firefighters who only sleep at home. Categorizing by age may also prove beneficial, as health conditions worsen with age.

With further research, interventions such as replacing bedding and pillows and cooling the barracks as much as possible could be implemented to improve sleep quality. Providing resources on how to increase exercise, improve eating habits, and live an overall healthier lifestyle are other options. This research could help illustrate the need for increased funding to help support the health of our first responders.

## Figures and Tables

**Table 1:** Firefighter sleep data. N = 10, age range = 18–55 years.

Shift	Total Sleep Time (h)	Sleep Efficiency (%)	WASO (min)	N1 (h)	N2 (h)	Slow Wave Sleep (h)	REM Sleep (h)	Sleep Spindles (Hz) *	Cortical Arousals (#/h) *	Autonomic Activations (#/h) *
Barracks	5.45	87.3	39.0	1.2	2.9	1.05	1.06	2.6	17.0	37.0
+/− SE	0.58	1.75	5.7	0.82	0.43	0.15	0.21	0.85	2.4	5.0
Home	5.71	88.4	24.0	0.47	2.8	1.17	1.07	11.0	12.0	24.0
+/− SE	0.52	0.91	4.4	0.08	0.38	0.17	0.15	2.9	1.7	3.4

The * denotes variables in which significance was found between groups.

**Table 2:** Normative sleep values [15]. Age range = 18–64 years.

Shift	Total Sleep Time (h)	Sleep Efficiency (%)	WASO (min)	N1 (h)	N2 (h)	Slow Wave Sleep (h)	REM (h)	Sleep Spindles (Hz)	Cortical Arousals (#/h)
Normative	7–9	70–100	0–45	0.35–0.45	3.5–4.5	1.4–1.8	1.75–2.25	9–15	0–20

## Data Availability

Data will be available upon request to the corresponding author through email.

## References

[1] Tempesta D., Socci V., De Gennaro L., Ferrara M. (2018). Sleep and emotional processing. Sleep Med. Rev..

[2] Worley S.L. (2018). The Extraordinary Importance of Sleep. Pharm. Ther..

[3] Czeisler C.A. (2015). Duration, timing and quality of sleep are each vital for health, performance and safety. Sleep Health.

[4] Yetton B.D., McDevitt E.A., Cellini N., Shelton C., Mednick S.C. (2018). Quantifying sleep architecture dynamics and individual differences using big data and Bayesian networks. PLoS ONE.

[5] Waterhouse J., Fukuda Y., Morita T. (2012). Daily rhythms of the sleep-wake cycle. J. Physiol. Anthropol..

[6] Watson N.F., Badr M.S., Belenky G., Bliwise D.L., Buxton O.M., Buysse D., Dinges D.F., Gangwisch J., Grandner M.A., Kushida C. (2015). Recommended Amount of Sleep for a Healthy Adult: A Joint Consensus Statement of the American Academy of Sleep Medicine and Sleep Research Society. Sleep.

[7] What Happens When You Sleep: The Science of Sleep. Sleep Foundation. Published 1 January 1970. https://www.sleepfoundation.org/how-sleep-works/what-happens-when-you-sleep.

[8] Campbell I.G. (2009). EEG Recording and Analysis for Sleep Research. Curr. Protoc. Neurosci..

[9] Potter G.D., Skene D.J., Arendt J., Cade J.E., Grant P.J., Hardie L.J. (2016). Circadian Rhythm and Sleep Disruption: Causes, Metabolic Consequences, and Countermeasures. Endocr. Rev..

[10] Wickwire E.M., Geiger-Brown J., Scharf S.M., Drake C.L. (2017). Shift Work and Shift Work Sleep Disorder: Clinical and Organizational Perspectives. Chest.

[11] Rodrigues S., Paiva J.S., Dias D., Cunha J.P.S. (2018). Stress among on-duty firefighters: An ambulatory assessment study. PeerJ.

[12] Stout J.W., Beidel D.C., Brush D., Bowers C. (2021). Sleep disturbance and cognitive functioning among firefighters. J. Health Psychol..

[13] Abbasi M., Rajabi M., Yazdi Z., Shafikhani A.A. (2018). Factors affecting sleep quality in firefighters. Sleep Hypn..

[14] Billings J., Focht W. (2016). Firefighter Shift Schedules Affect Sleep Quality. J. Occup. Environ. Med..

[15] Shrivastava D., Jung S., Saadat M., Sirohi R., Crewson K. (2014). How to interpret the results of a sleep study. J. Community Hosp. Intern. Med. Perspect..

[16] Lucey B.P., Mcleland J.S., Toedebusch C.D., Boyd J., Morris J.C., Landsness E.C., Yamada K., Holtzman D.M. (2016). Comparison of a single-channel EEG sleep study to polysomnography. J. Sleep Res..

[17] Levendowski D.J., Walsh C.M., Boeve B.F., Tsuang D., Hamilton J.M., Salat D., Berka C., Lee-Iannotti J.K., Shprecher D., Westbrook P.R. (2022). Non-rem sleep with hypertonia in Parkinsonian Spectrum Disorders: A pilot investigation. Sleep Med..

[18] Eric S., Nilong V. Wakefulness after Sleep Onset. Sleep Foundation. https://www.sleepfoundation.org/sleep-studies/wakefulness-after-sleep-onset#:~:text=Wakefulness%20after%20sleep%20onset%20is,their%20WASO%20is%2025%20minutes.

[19] Purcell S.M., Manoach D.S., Demanuele C., Cade B.E., Mariani S., Cox R., Panagiotaropoulou G., Saxena R., Pan J.Q., Smoller J.W. (2017). Characterizing sleep spindles in 11,630 individuals from the National Sleep Research Resource. Nat. Commun..

[20] Gais S., Mölle M., Helms K., Born J. (2002). Learning-dependent increases in sleep spindle density. J. Neurosci. Off. J. Soc. Neurosci..

[21] Janackova S., Sforza E. (2008). Neurobiology of sleep fragmentation: Cortical and autonomic markers of sleep disorders. Curr. Pharm. Des..

[22] Amatoury J., Azarbarzin A., Younes M., Jordan A.S., Wellman A., Eckert D.J. (2016). Arousal Intensity is a Distinct Pathophysiological Trait in Obstructive Sleep Apnea. Sleep.

[23] Huang G., Lee T.Y., Banda K.J., Pien L.C., Jen H.J., Chen R., Liu D., Hsiao S.-T.S., Chou K.-R. (2022). Prevalence of sleep disorders among first responders for medical emergencies: A meta-analysis. J. Glob. Health.

[24] Hartmann S., Ferri R., Bruni O., Baumert M. (2021). Causality of cortical and cardiovascular activity during cyclic alternating pattern in non-rapid eye movement sleep. Philos. Transact. A Math. Phys. Eng. Sci..

[25] Kleiber K., Smith C.J., Beck S.D., Hege A., Corgan M., West C.A., Hunnicutt L., Collier S.R. (2023). Familiarization with ambulatory sleep and blood pressure monitoring is necessary for representative data collection. Physiol. Rep..

